# Interplay between SpaO variants shapes the architecture of the *Salmonella* type III secretion sorting platform

**DOI:** 10.1128/mbio.00155-26

**Published:** 2026-02-27

**Authors:** José Eduardo Soto, Tingting Wang, Jorge E. Galán, Maria Lara-Tejero

**Affiliations:** 1 Department of Microbial Pathogenesis, Yale University School of Medicine 537605, New Haven, Connecticut, USA; University of Utah, Salt Lake City, Utah, USA

**Keywords:** type III secretion system, *Salmonella enterica*, bacterial pathogenesis, protein secretion

## Abstract

*Salmonella enterica* utilizes a virulence-associated type III secretion system (T3SS) to inject bacterial effectors directly into host cells. Central to this machinery is the sorting platform (SP), a cytosolic assembly whose core scaffolding protein, SpaO, is produced in two isoforms: a full-length (SpaO^L^) and a shorter variant (SpaO^short^) comprising the C-terminal 101 residues of SpaO^L^. Although SpaO^short^ is evolutionarily conserved across type III secretion systems, its precise function has remained elusive. Here, we combined a sensitive, real-time translocation assay with site-directed photo-crosslinking to inform the role of SpaO^short^ in *Salmonella* SPI-1 T3SS. Quantitative translocation data show that while SpaO^short^ is not absolutely required for effector translocation, its absence significantly dampens T3SS-mediated protein delivery. Biochemical and structural probing further defined the interfaces between SpaO^L^ and SpaO^short^, uncovering a previously unrecognized interaction mode between the two isoforms. Photo-crosslinking revealed that a single SpaO^L^ molecule accommodates a SpaO^short^ dimer via an N-terminal “docking motif,” an interaction that occurs *in vivo* while SpaO^L^ is associated with other SP components. These results support a model in which SpaO^short^ is integrated into the SP pods alongside SpaO^L^, OrgA, and OrgB, likely contributing to pod stabilization. Collectively, these findings provide new insights into how *Salmonella* and related bacteria assemble and maintain these specialized protein-injection systems.

**IMPORTANCE:**

*Salmonella enterica* is an increasing global public health threat. As part of its virulence arsenal, *Salmonella* relies on a type III secretion system (T3SS) or injectisome, a molecular injection device that translocates effector proteins into host cells to promote invasion and inflammation. A central component of this machine is the SpaO protein, which is produced in two forms: a full-length form and a shorter variant. Here, by studying the functional and structural relationship between the two SpaO forms in their native cellular environment, we define how and when they assemble within the injectisome. Employing quantitative injection assays in cultured cells, we define the shorter SpaO variant as an accessory structural piece that boosts effector delivery. These findings refine our understanding of injectisome assembly and function and provide mechanistic insight to inform future efforts to target T3SS-dependent pathogens through antivirulence strategies.

## INTRODUCTION

*Salmonella enterica* remains a leading cause of acute gastroenteritis worldwide, posing a significant public health burden particularly in low- and middle-income countries (1). Its virulence is largely driven by the type III secretion system (T3SS), a complex molecular device that delivers effector proteins into target eukaryotic cells (2, 3). The T3SS, or injectisome, forms a translocation conduit enabling effector proteins to move from the bacterial cytoplasm into the host cell. *Salmonella* pathogenesis requires the sequential action of two distinct T3SSs encoded within the *Salmonella* pathogenicity islands 1 (SPI-1) (4, 5) and 2 (SPI-2) (6). The SPI-1 T3SS mediates early host cell invasion and inflammation, whereas the SPI-2 T3SS supports intracellular survival and replication.

From an evolutionary standpoint, virulence-associated T3SSs are believed to have emerged through exaptation of an ancestral flagellar system, wherein motility-dedicated components were repurposed for protein injection (7). Accordingly, the virulence T3SS shares many structural and mechanistic features with the bacterial flagellar system (8). This evolutionary adaptation has been independently acquired by many other important pathogens such as *Shigella* spp., *Yersinia* spp., and *Pseudomonas* spp., and confers a competitive advantage by allowing these organisms to interact intimately with eukaryotic host cells (Table S1).

The T3SS architecture comprises two key structural modules: an envelope-embedded needle complex (NC) and a large cytosolic assembly known as the sorting platform (SP). The NC is a supramolecular structure built of stacked membrane rings anchoring a hollow needle-like projection that extends into the extracellular space (9). Within the NC, the export apparatus (10), a conical protein assembly partially embedded in the inner membrane, helps form a continuous channel for client protein transport (11, 12). Attached to the cytosolic side of the NC is the SP (13, 14). While the NC provides a conduit for effector passage through the bacterial envelope, the SP functions as a hub that orchestrates intracellular processes required for protein injection. Notably, the SP selects, sorts, and initiates client proteins destined for the T3SS pathway, ensuring efficient and timely delivery of effectors into host cells (13).

Recent *in situ* cryo-electron tomography studies in *Shigella* (15) and *Salmonella* (16) have revealed that the SP adopts an intricate, cage-like structure composed of five distinct proteins, each with its own stoichiometry. Together, these proteins form six “pods” arranged radially around a central hub. Each pod connects to the NC via the adaptor protein OrgA, while the SP’s core protein, SpaO, binds OrgA through its N-terminal domain, forming the pod’s main body. At the base of each pod, an OrgB dimer attaches to the SpaO C-terminus and extends inward to create a cradle for an InvC hexamer, which serves as the ATPase responsible for chaperone release from cognate secreted proteins (17). This chamber-like enclosure is thought to create a privileged compartment for client protein sorting and unfolding (14). Although the flagellar system also contains a homologous cytosolic structure known as the C-ring, its architecture is markedly different from that of the SP (18). Whereas the SP forms a partially open cage-like structure, the flagellar C-ring is a closed structure, likely reflecting its role in torque transmission.

SpaO is the primary constituent of the SP in *Salmonella*. This 303-amino-acid protein acts as a central hub for protein-protein interactions within the SP (19, 20), promoting the formation of the pods. Structurally, SpaO consists of an N-terminal region comprising the first third of the protein, which lacks experimental structural data but is predicted by AlphaFold to form a globular domain, followed by two surface presentation of antigens domains (SPOA1 and SPOA2). These domains adopt a domain-swapped arrangement critical for numerous protein-protein interactions (21–23). A unique and conserved feature of the *spaO* gene, and its homologs in other T3SS-containing bacteria, is that it encodes two distinct products: the full-length SpaO (SpaO^L^) and a shorter isoform (SpaO^short^) comprising the last 101 amino acids. The latter is generated from an internal translation initiation site located at the terminal third of the gene (20, 23). The presence of a similar short isoform in *Shigella* (21), *Yersinia* (22), *Xanthomonas* (24), and in the *Salmonella* SPI-2 T3SS (25), strongly suggests that SpaO^short^ plays an important role in T3SS function. Unlike SpaO^L^, SpaO^short^ contains only the SPOA2 domain (23), suggesting a function distinct from that of the full-length isoform.

Previous *in vitro* studies demonstrated that the SpaO^short^ variant forms tightly intertwined homodimers which in turn bind SpaO^L^ in a 2:1 stoichiometric ratio (two SpaO^short^ per one SpaO^L^) (21–23, 26). However, the molecular details of this heterocomplex—such as its architecture and the stage of sorting platform assembly at which it occurs—remain largely unknown. Likewise, although diffraction-limited imaging in *Yersinia* has shown co-localization of both isoforms and that SpaO^short^ is required for SpaO^L^ foci formation (27), it has remained unclear whether SpaO^short^ serves as an integral structural component of the sorting platform or whether it becomes dispensable once SpaO^L^ is incorporated into the SP. Conflicting data from *in vitro* functional assays (20–22, 25, 27) have further complicated efforts to pinpoint the specific contribution of SpaO^short^ to injectisome assembly and function.

To shed light on the elusive function of SpaO^short^, we investigated its functional and structural relationship with SpaO^L^. Using a highly sensitive split NanoLuc-based assay, we quantitatively show that the absence of SpaO^short^ impairs, but does not abolish, T3SS functionality, suggesting an ancillary yet critical role for SpaO^short^ in T3SS activity. Through extensive *in vivo* crosslinking, we uncovered previously unknown interfaces between the two SpaO isoforms, identifying a key structural motif at the SpaO^L^ N-terminus essential for recruiting SpaO^short^. Further *in vivo* protein-interaction studies reveal that SpaO^L^ possesses multiple, non-mutually exclusive binding sites for its partners, findings that have major implications for current structural models of the SP. Together, the integration of quantitative translocation assays with *in vivo* interaction mapping provides new insights into SpaO^short^’s role, clarifying its true contribution to SP formation and T3SS activity. Overall, this work advances our understanding of how SpaO^short^, an elusive SP component, integrates into both the architecture and function of the T3SS.

## RESULTS

### SpaO^short^ is required for efficient T3SS-mediated protein translocation

Despite the broad conservation of the short SpaO isoform among T3SSs, its functional contribution remains debated, as studies have reported a wide range of phenotypes across species. Previous work from our group and others showed that deletion of SpaO^short^ in *Salmonella* SPI-1 results in only mild defects in T3 secretion and host cell invasion (20, 26). Similarly, T3SS-mediated hemolysis assays in Shigella indicated residual, albeit detectable, T3SS activity in the absence of the SpaO^short^ homolog (28). In contrast, conventional *in vitro* assays have suggested that the short SpaO isoform is strictly essential for T3SS activity in Yersinia (22, 27) and Shigella (21). Therefore, to better define the functional role of SpaO^short^ in T3SS activity, we sought a method that would not only offer a broader dynamic range and higher sensitivity than standard *in vitro* secretion assays but would also closely mimic the physiological context in which Salmonella infects host cells.

**Fig 1:**
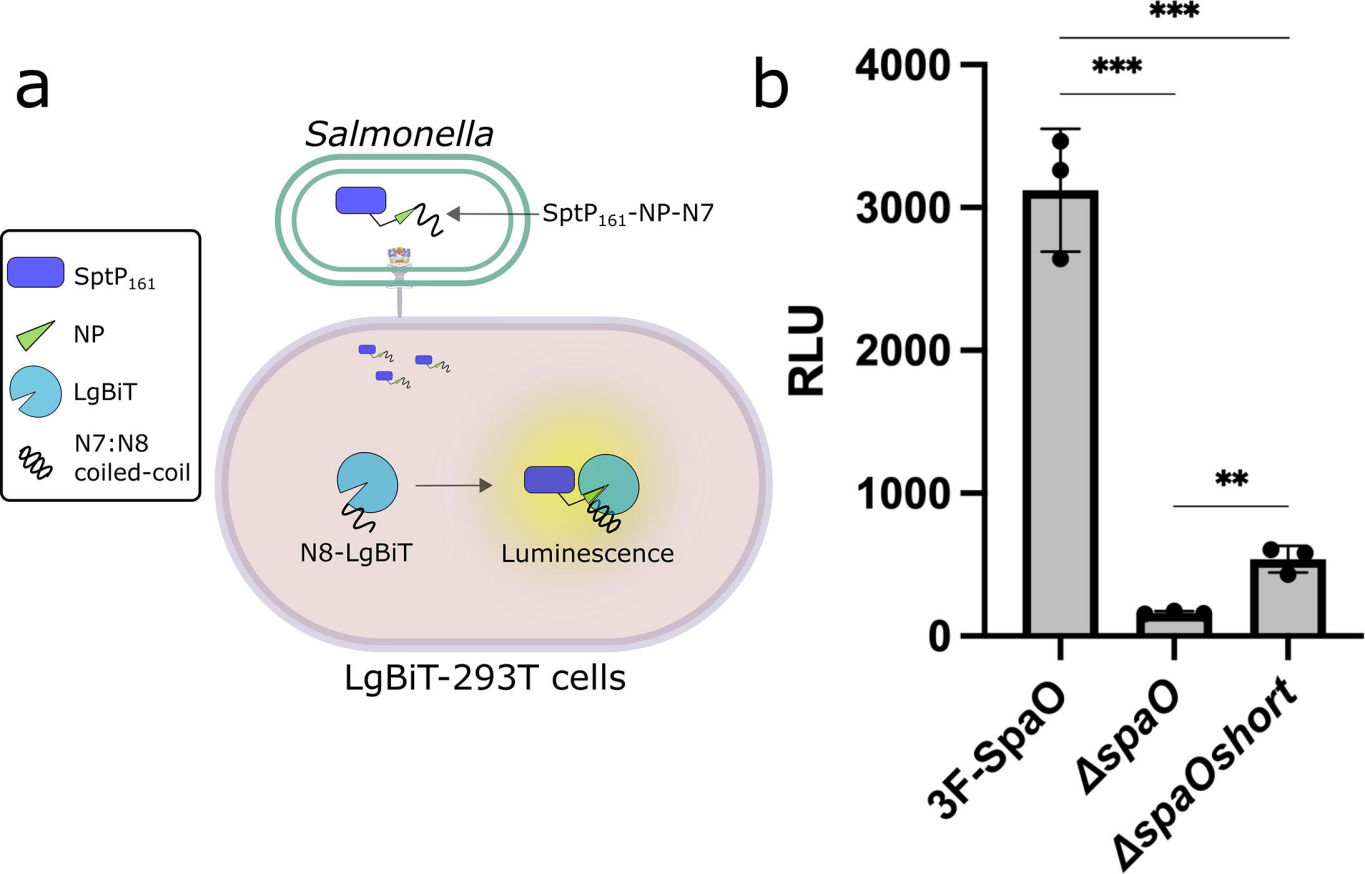
SpaO^short^ is required for fully functional T3SS activity. (**a**) Schematic diagram of the split NanoLuc-based translocation assay. *S*. Typhimurium was engineered to produce the first 161 residues of the SptP effector fused at its C-terminus to the NanoLuc peptide NP and the high-affinity peptide N7. Host cells stably producing the LgBiT moiety fused to the peptide N8 were infected, and T3SS-dependent translocation of the SptP-NP-N7 effector was detected as a luminescent signal following reconstitution of the luciferase reporter. (**b**) Luminescent signal resulting from the translocation of the SptP-NP-N7 effector into LgBiT-293T cells upon infection with wild-type *S*. Typhimurium or the otherwise isogenic Δ*spaO* or Δ*spaO^short^* strains. Data expressed as relative light units (RLU) are presented as means ± standard deviation. Statistical significance was evaluated using a paired Student’s *t*-test; ***P ≤ 0.001; **P ≤ 0.01.

We therefore adopted a split-nanoLuciferase assay (29), which allows quantitative, real-time measurement of effector translocation into cultured cells (30, 31). To implement this assay, we engineered a stable 293T cell line expressing the large fragment of NanoLuc luciferase (LgBiT), which lacks a single β-strand critical for its function (herein referred to as LgBiT-293T). Concurrently, we generated a construct in which the missing β-strand fragment (NP) was fused to the C-terminus of the first 161 aa of the SPI-1 effector SptP (SptP_161_-NP), which includes the secretion signal and the chaperone-binding domain (32), and co-expressed this fusion together with its cognate chaperone SicP in *Salmonella*. Functional reconstitution of NanoLuc occurs only if SptP-NP is translocated into host cells via the T3SS, where the NP fragment can complement LgBiT and restore luciferase activity (Fig. 1a). To improve reconstitution kinetics of the split luciferase, we appended the coiled-coil dimer-forming peptides N7 and N8, whose affinities lie in the low nanomolar range (33), to the NP and LgBiT moieties, respectively. The resulting SptP-NP-N7 fusion was expressed from an arabinose-inducible plasmid either in wild-type *S*. Typhimurium, the T3SS-deficient strain Δ*spaO* (negative control), or the Δ*spaO^short^* strain. In the Δ*spaO^short^* strain, the internal start codon (GTG_203) for *spaO^short^* is mutated to GCG in the *Salmonella* chromosome, thereby specifically preventing translation of the C-terminal SpaO^short^ isoform without affecting expression of SpaO^L^ (20).

LgBiT-293T cells were infected with these strains at a multiplicity of infection of 1:100 for 1 h, allowing T3SS-mediated translocation of SptP-NP-N7. Gentamicin was then added to eliminate extracellular bacteria, followed by washing and cell lysis for luminescence measurements. When infected with the wild-type strain, LgBiT-293T cells produced a robust luciferase signal (Fig. 1b). By contrast, the luciferase signal was drastically reduced in cells infected with the T3SS-deficient Δ*spaO* strain, confirming the specificity and dynamic range of the assay. Notably, infection with the Δ*spaO^short^* strain yielded a ~5-fold reduction in signal relative to wild type yet still exceeded the negative control signal. These results indicate that although the absence of SpaO^short^ impairs T3SS function, it does not abolish translocation altogether. These findings support the view that SpaO^short^, while not essential, plays a pivotal role in ensuring efficient T3SS-mediated effector delivery, and that the observed ~80% reduction in translocation activity more accurately reflects the extent to which loss of SpaO^short^ compromises T3SS activity.

### *In vivo* crosslinking defines the interface architecture of the SpaO^L^-SpaO^short^ complex within the sorting platform

**Fig 2:**
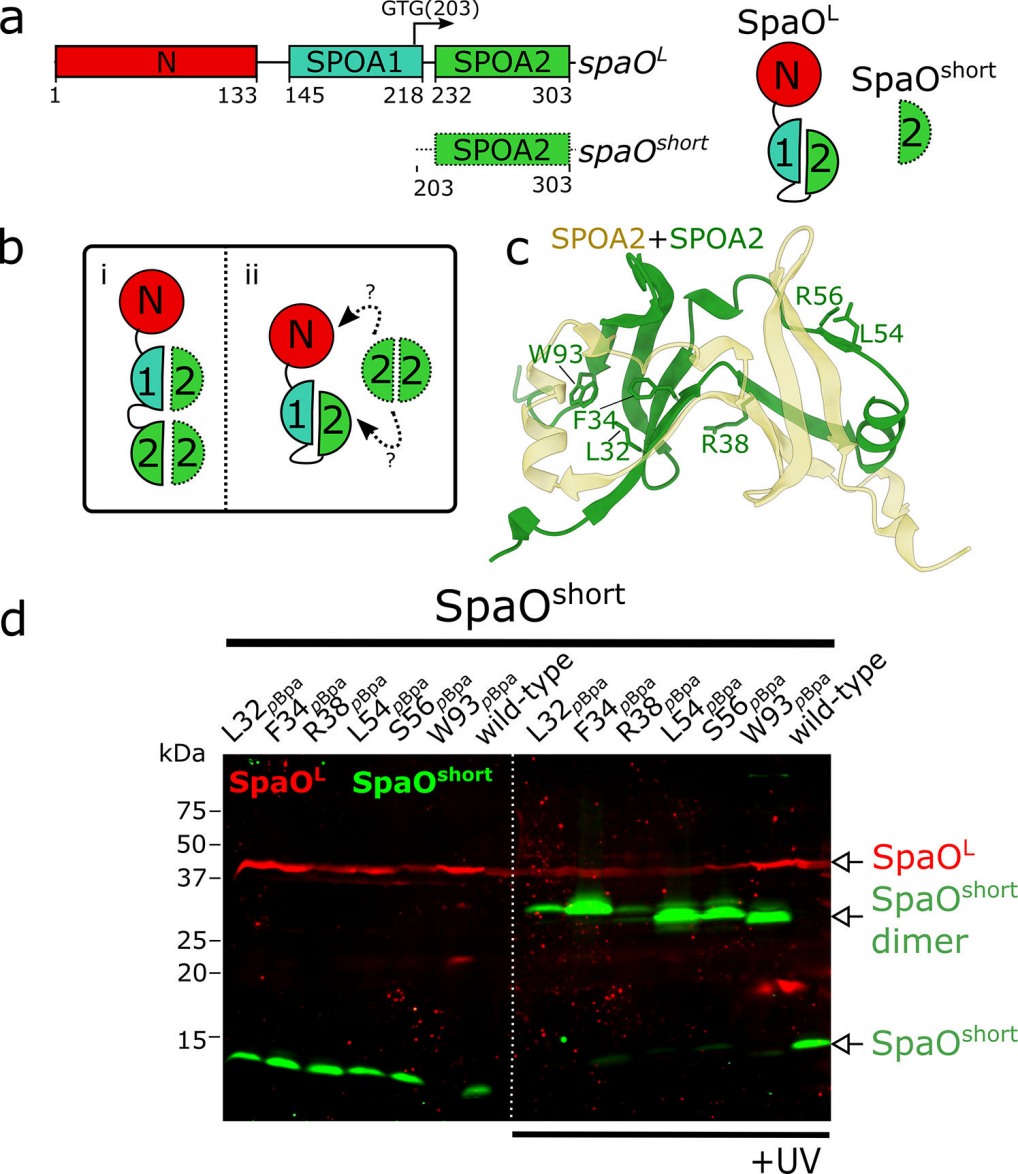
The SPOA2-SPOA2 interface of SpaO^short^ is exclusively committed to dimer formation. (**a**) Left: domain organization of *spaO^L^* and *spaO^short^*. The predicted N-terminal domain (red) and the corresponding SPOA1 and SPOA2 domains are indicated. The internal translation site at codon 203 that gives rise to the production of SpaO^short^ is marked by an arrow. Right: the SpaO^L^ protein is shown with its N-terminal domain followed by two covalently linked SPOA domains. SpaO^short^ comprises only the SPOA2 domain. (**b**) Potential interaction models between SpaO^L^ and SpaO^short^. In scenario i, either the SPOA1 or the SPOA2 domain of SpaO^L^ interacts with the SPOA2 domain of SpaO^short^ through the same interface used for SpaO^short^ dimerization. In scenario ii, the SpaO^short^ dimer binds SpaO^L^ using a distinct interaction mode, independent of the intertwined SPOA dimer interface. (**c**) Solved structure of the SPOA2 homodimer (PDB 4YX1), highlighting the six residues within a SpaO^short^ protomer (green) at the SPOA-SPOA interface that were individually replaced by *p*Bpa for crosslinking analysis. (**d**) Immuno-codetection of ^3FLAG^SpaO^L^ (red) and M45-SpaO^short^ (green) from *S*. Typhimurium strains expressing either wild-type proteins as controls or the indicated SpaO^short^ *p*Bpa mutant from its native chromosomal locus. Samples were left untreated (left) or exposed to UV light (right) to induce UV-dependent crosslinking. The detection of the SpaO^short^ crosslinked dimer is indicated.

The observed phenotype of the Δ*spaO^short^* mutant prompted us to investigate how SpaO^short^ interacts physically with its binding partner SpaO^L^. Although direct interaction between SpaO^L^ and SpaO^short^ (or their respective homologs) has been extensively documented (20–26, 28), the precise binding mode remains unclear, as no structure has been solved for the SpaO^L^-SpaO^short^ complex or for any virulence-associated T3SS ortholog complex. SpaO^L^ contains two SPOA domains, SPOA1 (residues 140–218) and SPOA2 (residues 232–303) (Fig. 2a). In contrast, SpaO^short^ comprises solely the SPOA2 domain (Fig. 2a). *In vitro* experiments have shown that the SPOA domains can engage in homotypic (SPOA2-SPOA2) or heterotypic (SPOA2-SPOA1) interactions (23), suggesting the existence of potentially different SpaO^short^/SpaO^L^ interfaces *in vivo* (34). When and where these interfaces potentially form is unknown. Therefore, to clarify this issue, we investigated whether, during sorting platform assembly, SpaO^short^ engages in intermolecular interactions with the SPOA domains of SpaO^L^ (Fig. 2b).

To this end, we conducted a site-specific photo-crosslinking survey informed by the crystal structure of the SPOA2 homodimer (23). We used the UV-photoactivatable noncanonical amino acid *p*-benzoyl-L-phenylalanine (*p*Bpa), which can be genetically incorporated at any desired position by suppressing a UAG stop codon (35). Because *p*Bpa has a short reactive distance (~3.1 Å) (36) that is comparable to typical hydrogen-bond lengths (2.7–3.3 Å) (37), it forms a covalent crosslink only when inserted into actual protein-protein interfaces.

To specifically target SpaO^short^, we genetically uncoupled *spaO^short^* from *spaO^L^* by replacing the native *spaO* gene with a bicistronic construct coding for 3xFLAG epitope-tagged SpaO^L^ (3xFLAG-SpaO^L^) and a downstream, recoded M45 epitope-tagged SpaO^short^ (M45-SpaO^short^), as reported previously (20). Guided by the SPOA2 homodimer structure (PDB 4YX1), we introduced *p*Bpa at six distinct residues in SpaO^short^ (L32, F34, R38, L54, S56, W93; numbering based on M1 as the first residue of the alternative translation product) located at the dimeric interface (Fig. 2c). We reasoned that, if at any stage of SP assembly—either transiently in the cytosol or within the final SP complex—SpaO^short^ interacts with SpaO^L^, crosslinking in live cells would capture this association.

After verifying that SpaO^short^ UAG variants were suppressed in a *p*Bpa-dependent manner upon addition of the unnatural amino acid to the medium (Fig. S1a), UV irradiation of each *p*Bpa-containing strain produced a ~30 kDa crosslinked species consistent with SpaO^short^ dimer formation (Fig. 2d). However, no SpaO^short^-SpaO^L^ adduct was detected (Fig. 2d and Fig. S1b), suggesting that, *in vivo*, the homotypic SPOA2-SPOA2 interaction is exclusive to SpaO^short^ protomers (scenario ii in Fig. 2b) rather than occurring between SpaO^short^ and any of the SPOA domains of SpaO^L^ (scenario i in Fig. 2b).

**Fig 3:**
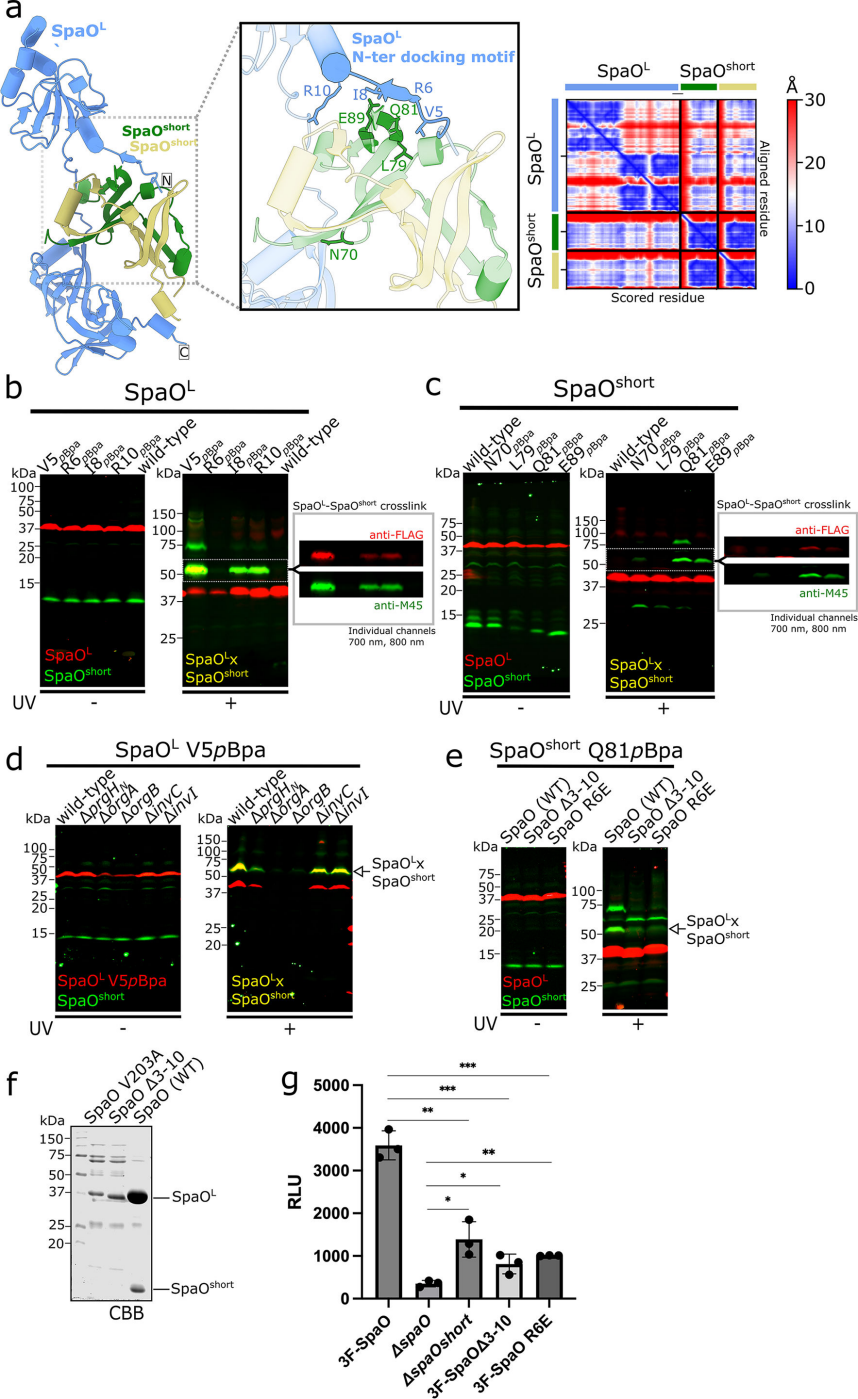
The N-terminal “docking motif” in SpaO^L^ mediates recruitment of SpaO^short^. (**a**) AlphaFold model of SpaO^L^ (blue) in complex with the SpaO^short^ dimer (green and khaki). (Inset, middle) Close-up view of the predicted docking motif at the SpaO^L^-SpaO^short^ interface. Interfacial residues that were substituted by *p*Bpa for crosslinking analysis are highlighted. The AlphaFold Predicted Alignment Error (PAE) plot on the right indicates the pairwise confidence in residue positioning, ranging from blue (high confidence) to red (low confidence). (**b and c**) Immuno-codetection of ^3FLAG^SpaO^L^ (red) and M45-SpaO^short^ (green) in *S*. Typhimurium strains expressing either wild-type proteins as controls or the indicated *p*Bpa-containing SpaO^L^ (**b**) or SpaO^short^ (**c**) from their native chromosomal loci. Samples were either left untreated (left) or exposed to UV light (right) to induce UV-dependent crosslinking. The detection of the SpaO^L^-SpaO^short^ crosslinked complex is indicated. Boxed insets show channel-separated Western blot signals reactive to both anti-FLAG and anti-M45 antibodies, corresponding to the indicated crosslinked adducts. (**d**) Whole-cell lysates of *S*. Typhimurium wild-type or the indicated isogenic mutant strain chromosomally expressing ^3FLAG^SpaO^L^ V5pBpa and M45-SpaO^short^, exposed to UV light or left untreated, were analyzed by Western blot. The detection of the SpaO^L^-SpaO^short^ crosslinked complex is indicated. (**e**) Whole-cell lysates of *S*. Typhimurium strains chromosomally expressing M45-SpaO^short^ (green) carrying *p*Bpa at position Q81 (green) and ^3FLAG^SpaO^L^ (red), either wild-type or the indicated mutant derivative. Samples were left untreated (left) or exposed to UV light (right) to induce UV-dependent crosslinking. The detection of the SpaO^L^-SpaO^short^ crosslinked complex is indicated. (**f**) N-terminal His-tagged wild-type SpaO or its mutant derivatives SpaOV203A (lacking the internal translation site), or SpaOΔ3-10 (lacking the N-terminal docking motif), were expressed in *E. coli*, purified by Ni-NTA resin, and visualized by Coomassie brilliant blue staining. His-SpaO^L^ and untagged SpaO^short^ are indicated. (**g**) Luminescent signal resulting from the translocation of the SptP-NP-N7 effector into LgBiT-293T cells upon infection with wild-type *S*. Typhimurium chromosomally expressing ^3FLAG^SpaO or the otherwise isogenic Δ*spaO*, Δ*spaO^short^*, ^3FLAG^SpaOΔ3-10, or ^3FLAG^SpaO R6E mutant strains. Data expressed as RLU are presented as means ± standard deviation. Relevant statistical significance was evaluated using a paired Student’s *t*-test; ***P ≤ 0.001; **P ≤ 0.01; *P ≤ 0.05.

Earlier work indicated that, in solution, the SpaO^short^ dimer associates with a single SpaO^L^, resulting in the formation of SpaO^L^-2SpaO^short^ heterotrimer, which can potentially make up the basic building block of each “pod” within the SP (22, 26). Although it has been previously alternatively suggested that SpaO^L^ engages the SpaO^short^ dimer through either its N- (26) or C- terminal region (20, 25), the precise configuration of this complex has been unclear. To obtain structural insights into the specific arrangement of SpaO^L^ and the SpaO^short^ homodimer complex, we leveraged AlphaFold (38, 39), which predicted that the first 10 residues of SpaO^L^ anchor the SpaO^short^ dimer through a “docking motif” (Fig. 3a). Specifically, residues 6–8 of SpaO^L^ are predicted to adopt a β-strand conformation when bound to the SpaO^short^ dimer, which aligns with the fifth β-strand of one SpaO^short^ protomer (Fig. 3a, inset). This N-terminal extreme β-strand is not predicted to form when SpaO^L^ is modeled alone, suggesting that SpaO^short^ binding induces SpaO^L^ to adopt this conformation (Fig. 3a, right and Fig. S2).

To validate the predicted spatial proximity between the SpaO^L^ N-terminal β-strand (i.e., the “docking motif”) and SpaO^short^, we replaced in the *S*. Typhimurium chromosome four SpaO^L^ residues (V5, R6, I8, and R10) with *p*Bpa in the uncoupled 3xFLAG-SpaO^L^ M45-SpaO^short^ strain. Following UV irradiation of live bacteria, we analyzed crosslinked products by SDS-PAGE and immunoblotting with the anti-FLAG and anti-M45 antibodies. In the negative-control strain (i.e., wild type), which lacks *p*Bpa substitutions, no crosslinks were observed upon UV exposure. However, three out of the four *p*Bpa variants (V5, I8, and R10) produced a robust ~50 kDa adduct consistent with a SpaO^L^-SpaO^short^ crosslinked complex (Fig. 3b). Fluorescent channel splitting confirmed that the ~50 kDa species contained both SpaO^L^ and SpaO^short^, demonstrating successful capture of the SpaO^L^-SpaO^short^ complex *in vivo*. Similar crosslinking patterns were detected when the *p*Bpa substitutions were introduced in the native *spaO* locus (i.e., producing SpaO^L^ and SpaO^short^ from a single cistron; Fig. S3), ruling out potential artifacts caused by genetic uncoupling. We noted a ~75 kDa UV-dependent band in the V5^pBpa^ mutant and in some SpaO^short-pBpa^ substitutions, which we interpret to represent the presence of aberrant migration forms of crosslinked products.

Next, to pinpoint SpaO^short^ residues that interface with SpaO^L^, we carried out “reverse” photo-crosslinking experiments using four surface-exposed SpaO^short^ residues (N70, L79, Q81, E89) that AlphaFold predicts to be in close spatial proximity to SpaO^L^. UV exposure of *p*Bpa-substituted SpaO^short^ variants resulted in the formation of a ~50 kDa adduct corresponding to SpaO^L^-SpaO^short^, most prominently in Q81^pBpa^ and E89^pBpa^ (Fig. 3c). We also observed a weaker but detectable crosslink in the N70^pBpa^. In the AlphaFold model, Q81 of one SpaO^short^ protomer lies near the docking motif on SpaO^L^ (Fig. 3a, inset), consistent with the strong crosslinking signal. Like what we observed in the V5^pBpa^-SpaO^L^ crosslinks, an additional ~75 kDa band was detected in the crosslinks of Q81^pBpa^–SpaO^short^, reinforcing the conclusion that these adducts represent the same complex with altered electrophoretic mobility. Altogether, these data refine the SpaO^L^-SpaO^short^ docking interface and establish the N-terminal region of SpaO^L^ as a key binding site for SpaO^short^ within the SP.

With robust crosslink “reporter” sites in hand, we next explored whether formation of the SpaO^L^-SpaO^short^ complex depends on other SP components or a fully assembled T3SS. Introducing the 3×FLAG–SpaO^L-V5pBpa^ allele into strains lacking the ATPase InvC or the stalk protein InvI did not affect the crosslinking patterns (Fig. 3d). Since these mutations do not alter SP assembly but render the injectisome non-functional (16), these results indicate that the SpaO^L^-SpaO^short^ interaction is independent of an active injectisome. Interestingly, deleting the cytosolic domain of PrgH, which leaves the SP fully cytoplasmic by abrogating its membrane anchor, only mildly reduced SpaO^L^-SpaO^short^ crosslinking relative to the wild-type background (Fig. 3d). In contrast, *orgA* or *orgB* deletions destabilized SpaO^L-V5pBpa^, hindering assessment of the crosslink in those strains. Collectively, these results indicate that SpaO^short^ recruitment by SpaO^L^ occurs prior to the SP’s docking into the needle complex and does not require an intact, functional injectisome.

### The N-terminal docking motif of SpaO^L^ is essential for the recruitment of SpaO^short^ to the sorting platform

To further dissect the functional role of the SpaO^L^ “docking motif,” we examined whether a truncated SpaO^L^ variant lacking residues 3–10 (SpaO^Δ3–10^) could still crosslink with SpaO^short-Q81pBpa^. Chromosomal deletion of residues 3–10 in *Salmonella* abolished both the ~50 kDa and ~75 kDa crosslinked adducts formed between SpaO^L^ and SpaO^short-Q81pBpa^ (Fig. 3e). This loss coincided with the appearance of a ~60 kDa band, which we hypothesize reflects misassembled SpaO^short^ oligomers. Similarly, a single point mutation in the docking motif itself, introducing a residue with opposite charge (SpaO^L-R6E^), was sufficient to disrupt crosslinking with SpaO^short-Q81pBpa^. These data confirm that SpaO^short^ targets the N-terminal β-strand (i.e., the “docking motif”) in SpaO^L^.

We next used *in vitro* pull-down assays to verify the necessity of the N-terminus “docking motif” of SpaO^L^ for SpaO^short^ recruitment. As reported previously (20), heterologous expression of N-terminal His-tagged SpaO (His-SpaO) in *E. coli* enables co-purification with SpaO^short^. By contrast, expressing His-SpaO^V203A^ (lacking the SpaO^short^ isoform due to mutation of its internal start codon) markedly reduced the amount of soluble His-SpaO^L^ (Fig. 3f). Notably, His-SpaO^Δ3–10^, which preserves the internal translation start site but lacks residues 3–10, similarly compromised SpaO^L^ solubility and prevented its co-purification with SpaO^short^ (Fig. 3f). These results underscore the pivotal role of the SpaO^L^ N-terminus “docking motif” in SpaO^short^ recruitment.

Because residues 3 to 10 of SpaO^L^ are critical for SpaO^short^ binding, we hypothesized that deletion of these residues should have a functional impact in T3SS activity. Using the split-nanoLuciferase–based assay, we quantified effector translocation in cells infected by an *S*. Typhimurium strain encoding *spaO^Δ3–10^* on the chromosome. Deletion of residues 3–10 in SpaO reduced SptP–NP–N7 translocation levels to the same extent observed in the *ΔspaO^short^* strain (Fig. 3g). In agreement with these results, the SpaO^R6E^ point mutant also exhibited similarly diminished translocation, again mirroring the absence of SpaO^short^. Overall, these findings establish the SpaO^L^ “docking motif” as a crucial determinant for SpaO^short^ recruitment and underscore its essential role in T3SS function.

### Dissection of the modular architecture of the SpaO^L^-SpaO^short^ complex within the sorting platform

SpaO^L^ is the structural scaffold of the *Salmonella* T3SS sorting platform (SP) and acts as a hub for the protein-protein interactions that assemble this megadalton-size complex (16). Its N-terminal domain binds the symmetry adaptor OrgA (40), whereas the intertwined SPOA1–SPOA2 C-terminal region docks into the OrgB “cradle” (20, 23, 40). However, the potential structural placement of SpaO^short^ within the SP remains unclear. Although *in vitro* co-purification experiments have yielded SpaO^L^-SpaO^short^ soluble complexes containing either OrgA (28) or OrgB (26), these assemblies could originate from distinct SpaO^L^ subpopulations, with some molecules binding SpaO^short^ and others interacting with OrgA/OrgB, rather than reflecting a single, physiologically relevant complex. Specifically, it is unknown whether SpaO^short^ is associated with the SpaO^L^ subpopulation engaged in pod formation (i.e., those that contact OrgA and OrgB).

**Fig 4:**
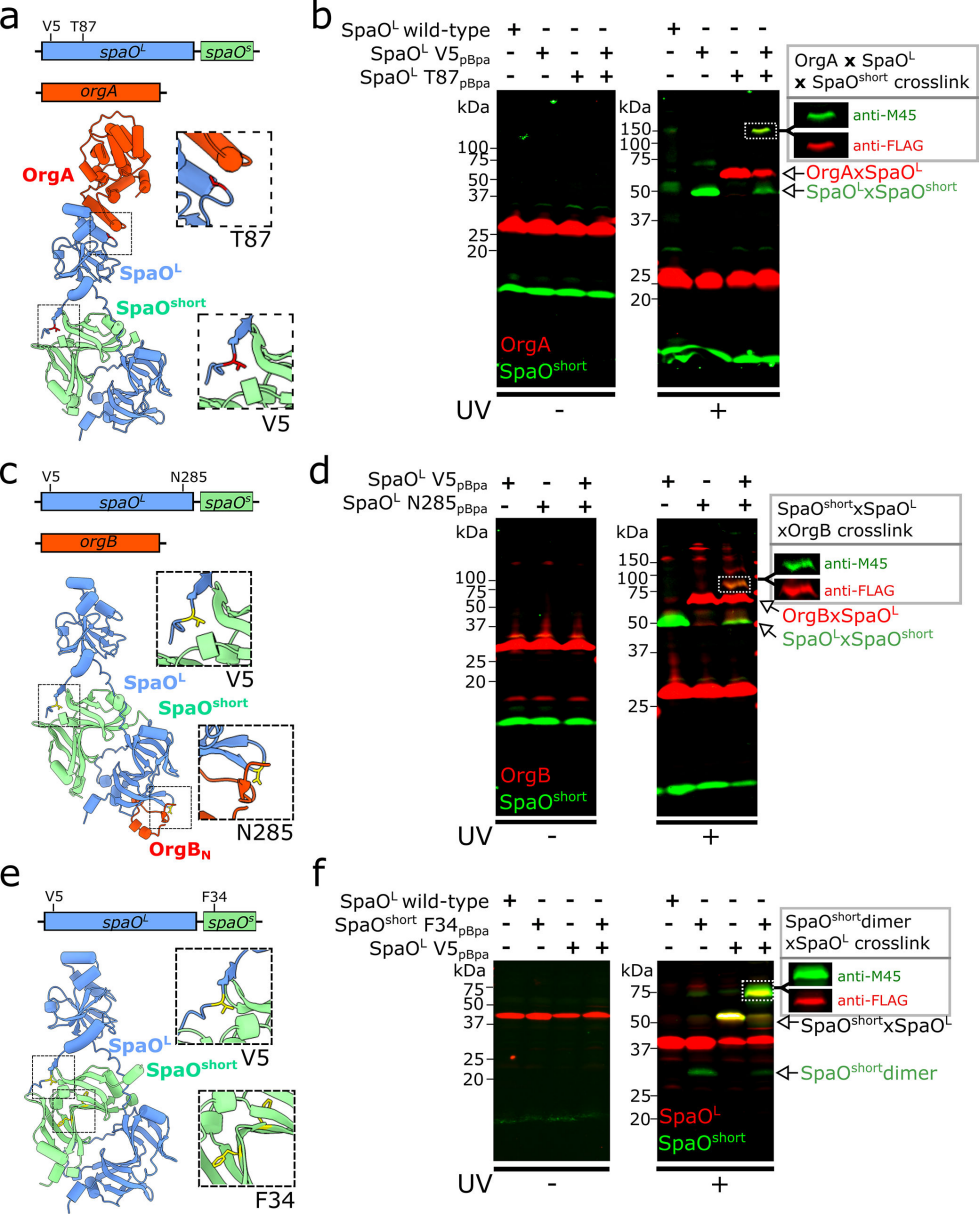
Dual photo-crosslinking reveals the modular architecture of SpaO^L^. (**a, c, and e**) AlphaFold models of the trimeric complexes: (**a**) OrgA-SpaO^L^-SpaO^short^, (**c**) OrgB_N_-SpaO^L^-SpaO^short^, and (**e**) SpaO^L^-SpaO^short^-SpaO^short^. For the sake of clarity, only the SpaO^L^-binding region of OrgB (OrgB_N_, residues 5–34) is shown in panel **c**. Residues in SpaO^L^ or SpaO^short^ that were substituted by *p*Bpa for photocrosslinking analysis are mapped onto the structure, with close-up views of these regions in the insets. (b, d, and f). Whole-cell lysates from *S*. Typhimurium strains chromosomally expressing (**b**) M45-SpaO^short^ (green), OrgA-3FLAG (red), and 2xHA-SpaO^L^ V5- and T87-*p*BpA; (**d**) M45-SpaO^short^ (green), OrgB-3FLAG (red), and 2xHA-SpaO^L^ V5- and N285-*p*BpA; (**f**) M45-SpaO^short^ (green) F34-*p*BpA (dimer reporter) and ^3FLAG^SpaO^L^ (red) V5-*p*Bpa. Samples were left untreated (left) or exposed to UV light (right) to induce UV-dependent crosslinking. The formation of single and double cross-linked adducts is indicated. Boxed insets show channel-separated Western blot signals reactive to both anti-FLAG and anti-M45 antibodies, highlighting the capture of the trimeric complexes.

To address this question, we used *in vivo* photo-crosslinking. The photoactivatable amino acid *p*Bpa was placed at SpaO^L^ residues previously shown to capture SpaO^L^-OrgA (SpaO^T87pBpa^) or SpaO^L^-OrgB (SpaO^N285pBpa^) interactions (40). These reporters were introduced into a strain that chromosomally expresses OrgA-3×FLAG and M45-SpaO^short^, together with a 2xHA-tagged SpaO^L^ variant carrying *p*Bpa simultaneously at V5 (reporting SpaO^L^-SpaO^short^) and T87 (reporting SpaO^L^-OrgA; Fig. 4a). UV irradiation of this “double *p*Bpa” SpaO^L^ yielded a high-molecular-weight crosslinked adduct that reacted with both anti-FLAG and anti-M45 antibodies, indicating it contained OrgA-3×FLAG and M45-SpaO^short^ (Fig. 4b). Although this band migrated more slowly than expected for a simple trimer, its formation strictly required the simultaneous presence of V5^pBpa^ and T87^pBpa^ in SpaO^L^, showing that a trimeric OrgA-SpaO^L^-SpaO^short^ complex had been captured. Control strains harboring only one *p*Bpa site in SpaO^L^ formed only the corresponding binary complexes (Fig. 4b).

We performed the same analysis with OrgB. A strain encoding 2xHA-SpaO^L^ with *p*Bpa at both V5 (SpaO^short^ binding reporter) and N285 (OrgB binding reporter) was chromosomally engineered to co-express OrgB-3×FLAG and M45-SpaO^short^ (Fig. 4c). UV exposure produced an ~80 kDa adduct that was recognized by both anti-FLAG and anti-M45 antibodies, consistent with a SpaO^L^-OrgB-SpaO^short^ trimer (Fig. 4d).

Together, these experiments demonstrate that a single SpaO^L^ molecule can bind SpaO^short^ while simultaneously engaging either OrgA or OrgB. The three interaction surfaces, SpaO^L^/SpaO^short^, SpaO^L^/OrgA, and SpaO^L^/OrgB, are therefore spatially distinct and non-overlapping.

Next, given that *in vitro* studies suggest that a SpaO^short^ dimer constitutes the unit that engages a single SpaO^L^ (21, 26), we sought to formally test whether the SpaO^short^ that we captured, recruited to the SpaO^L^ N-terminal “docking motif,” corresponds to this dimeric form. To this end, we engineered a strain chromosomally expressing M45–SpaO^short^ carrying F34*p*Bpa (which reports SpaO^short^ dimerization, Fig. 2d), along with 3xFLAG-SpaO^L^, carrying V5pBpa (which reports SpaO^L^-SpaO^short^ binding, Fig. 4e). Upon UV irradiation, we observed a ~70 kDa crosslinked adduct containing both M45-SpaO^short^ and 2xHA–SpaO^L^ (Fig. 4f). In agreement with our earlier experiments (Fig. 3d) and previous *in vitro* reports, this result is consistent with SpaO^L^ engaging a preformed SpaO^short^ dimer, supporting a model in which the SpaO^short^ dimer docks onto the N-terminal end of SpaO^L^ to assemble the functional sorting platform pods.

## DISCUSSION

In this work, we set out to clarify the elusive role of the shorter SpaO isoform (SpaO^short^) in the *Salmonella* SPI-1 type III secretion system. Although SpaO^short^ has long been known to coexist with its full-length counterpart (SpaO^L^) in many T3SS-containing bacteria, its precise function, whether as a structural component of the sorting platform (SP) or as a regulator of its assembly, has remained unresolved. By combining a highly sensitive *in vivo* translocation assay, extensive site-directed crosslinking, and structure-guided mutagenesis, we demonstrate that SpaO^short^ is a structural ancillary component of the sorting platform, directly engaging SpaO^L^ molecules that are themselves integrated into the SP.

A key finding of our study is that SpaO^short^, although not strictly required for T3SS activity, is nonetheless critical for efficient effector translocation into host cells. This observation helps reconcile discrepancies from early *in vitro* studies, where the impact of SpaO^short^ deletion on type III protein secretion appeared variable or modest, by employing a more physiologically relevant, real-time assay. Using our split nanoLuciferase assay, we found that *Salmonella* strains lacking SpaO^short^ still translocate effectors, but at substantially reduced levels compared to the wild type. These results support a model in which SpaO^short^ fine-tunes T3SS function, likely by contributing to the stability and/or efficiency of sorting platform assembly, thereby optimizing effector translocation. The complete loss of T3SS activity reported upon deletion of *spaO^short^* homologs in other T3SS-carrying bacteria (21, 22, 27) likely reflects methodological differences in assay sensitivity rather than genuine mechanistic divergences in this conserved small isoform.

Building on these functional insights, our photo-crosslinking survey exposed a previously unknown mode of interaction between SpaO^L^ and SpaO^short^. First, although the SPOA domain of SpaO^short^ could, in principle, engage in heterotypic interactions with the SPOA domains of SpaO^L^, we found no evidence for such contacts (Fig. 2), at least not through the canonical SPOA interface. Second, guided by AlphaFold and validated through *in vitro* and *in vivo* PPI assays and functional analyses, we mapped the SpaO^L^-SpaO^short^ interface, revealing a previously unappreciated docking motif at the extreme N-terminal end of SpaO^L^ that is strictly required for SpaO^short^ recruitment. Mutations or deletions that disrupt this docking interface prevent SpaO^L^-SpaO^short^ complex formation and impair T3SS-mediated translocation to the same extent as deleting SpaO^short^ outright, underscoring its functional importance. It is important to mention that, in a previous study, we detected a crosslink between the C-terminal residue L276 of SpaO^L^ and SpaO^short^ (20). Although the signal of such crosslink was modest, together with the data here presented, it supports a model in which the SpaO^short^ dimer is “sandwiched” between the N-terminal third of SpaO^L^ and its C-terminal SPOA1-SPOA2 domains (Fig. 3a and Fig. S4a). Notably, this arrangement parallels the recently described interaction between the *Salmonella* flagellar proteins FliM and FliN (41), where an N-terminal β-strand extension of FliM binds a FliN dimer (Fig. S4b). This similarity reinforces the evolutionary link between the T3SS sorting platform and the flagellar C-ring, suggesting that structural principles governing scaffold assembly may be conserved across these two systems.

These insights help to clarify why the injectisome SP and the flagellar C-ring diverge in the overall architecture. In the flagellar C-ring, the single C-terminal SPOA domain of FliM (SpaO^L^ counterpart) associates with three FliN (SpaO^short^ counterpart) subunits to form a heterotetrameric SPOA module. These SPOA modules interlock laterally to generate a continuous spiral that constitutes the bottom base of the C-ring, thereby enabling ring oligomerization. By contrast, in the injectisome, SpaO^short^ engages SpaO^L^, which, by virtue of its covalent linkage between the SPOA1 and SPOA2 domains, presents only capped SPOA surfaces and therefore interacts in a less intricate manner. Instead of promoting extensive inter-subunit connectivity, SpaO^short^ primarily stabilizes each of the six discrete SP pods (Fig. S4). This structural divergence aligns with functional requirements: SpaO^short^ serves as an ancillary stabilizer of SP pods, whereas FliN is strictly required for flagellar function (42).

Our data further demonstrate that SpaO^short^ associates with SpaO^L^ while SpaO^L^ is bound to other key SP components, such as OrgA or OrgB, indicating that these interfaces are not mutually exclusive. This observation directly supports a model in which a single SpaO^L^ molecule can simultaneously interact with multiple partners, reinforcing the concept of SpaO^L^ as a multivalent scaffold. Additionally, by dual-photo-crosslinking both SpaO^L^ and SpaO^short^, we captured a species consistent with a SpaO^L^-2(SpaO^short^) heterotrimer, confirming that SpaO^short^ is incorporated as a homodimer *in vivo*. Together, these findings highlight a sophisticated modular architecture in which SpaO^short^ integrates into the pods of the sorting platform alongside the established OrgA-OrgB-SpaO^L^ framework. Importantly, the incorporation of SpaO^short^ into SpaO^L^ is a critical determinant of SP pod integrity and, consequently, of efficient type III secretion. Given that the SP acts as the organizational hub for substrate recognition, processing, and delivery to the export gate, our results suggest that removal of SpaO^short^ from the pods leads to pronounced adverse effects on secretion efficiency. In this context, it will be particularly interesting to investigate how SpaO^short^ engagement contributes to the previously described sequential loading of substrates onto the SP (13), potentially by stabilizing specific conformational states or interaction networks required for ordered secretion.

Beyond expanding our basic understanding of T3SS assembly, these insights have several broader implications. First, they help reconcile conflicting phenotypes surrounding SpaO^short^ across different bacterial species by revealing that the shorter isoform fine-tunes effector delivery rather than serving as an essential component. Second, they suggest potential targets for rational therapeutic intervention: compounds that disrupt the hitherto uncovered SpaO^L^-SpaO^short^ interface could destabilize the entire sorting platform and attenuate virulence without fully inhibiting basic bacterial physiology. Third, our discovery of a conserved docking motif may inform future structural or functional comparisons between T3SSs and the ancestral flagellar system, illuminating how nature repurposes related molecular machinery for distinct roles in motility versus pathogenesis.

In summary, our work establishes SpaO^short^ as a stabilizing integral structural component of the *Salmonella* sorting platform rather than a regulatory factor. Through a combination of quantitative translocation assays and fine-grained crosslinking, we delineated a new docking motif in SpaO^L^ that recruits the SpaO^short^ homodimer. These findings provide a clearer picture of how the T3SS sorting platform is assembled, offering new perspectives on the molecular organization of this essential virulence system.

## MATERIALS AND METHODS

### Bacterial strains and plasmids

All strains used in this study are derivatives of *Salmonella enterica* serovar Typhimurium SL1344 (43) and are listed in Table S2. Bacterial strains were routinely grown in lysogenic broth (LB) at 37°C. For SPI-1 T3SS induction, *S*. Typhimurium was cultured in LB supplemented with 0.3 M NaCl under low-aeration conditions using a low-speed rotating wheel. Where indicated, expression of the SPI-1 master regulator HilA was induced from an arabinose-inducible plasmid (44). Selective antibiotics were used at the following concentrations (when required): streptomycin, 100 μg/mL; tetracycline, 10 μg/mL; ampicillin, 100 μg/mL; and chloramphenicol, 10 μg/mL.

Chromosomal modifications (in-frame gene deletions and insertions) were introduced by allelic exchange using the R6K-based suicide vector pSB890 (45) and *E. coli* β-2163 Δ*nic35* (46) as the conjugative donor strain. Plasmids for this work were constructed using Gibson assembly (47) and are listed in Table S2.

### Generation of stable cell lines

A HEK-293T cell line stably expressing the N8-LgBiT-HA fusion protein was generated by lentiviral transduction followed by puromycin selection, as described previously (48). Briefly, viruses were produced by co-transfecting HEK-293T cells with plasmids encoding N8-LgBiT-HA and the required packaging factors. After 48 h, the viral supernatant was harvested and used to infect HEK-293T cells. Transduced cells were then selected in 2 μg/mL puromycin (Gibco) for 5 days, and resistant cells were cloned into 96-well plates to obtain stable single-cell clones.

### Split NanoLuc-based translocation assay

To construct strains for the T3SS-dependent translocation assay, a fragment containing the *sicP* cognate chaperone followed by the first 161 codons of the SPI-1 effector SptP was amplified. The nanoluciferase small fragment (NP) plus the coiled-coil module N7 were fused in frame to the C-terminus of SptP_161_ (SptP-NP-N7). The fusion was cloned into pBAD24A and introduced into the *S. Typhimurium* strains of interest.

HEK-293T cells stably expressing N8-LgBiT-HA (LgBiT-293T) were seeded in 24-well plates and grown for 24 h prior to infection. Bacterial cultures, grown under T3SS-inducing conditions (LB + 0.3 M NaCl) with 0.1% arabinose, were added to the LgBiT–293T cells at a multiplicity of infection of 100 in Hank’s balanced salt solution (Gibco 14025092) containing calcium and magnesium. After a 1 h infection, cells were washed three times with prewarmed PBS and incubated in DMEM (10% bovine calf serum) supplemented with 50 μg/mL gentamicin for 1 h to kill extracellular bacteria. Following incubation, cells were washed three additional times with PBS and lysed in water (to avoid lysing intracellular bacteria). Lysates were clarified by centrifugation (14,000 × *g* for 5 min), and 20 μL of the supernatant was transferred to 96-well black-wall plates (Costar). Luciferase activity was measured using the Nano-Glo Luciferase Assay System (Promega) by adding 20 μL of the Nano-Glo working solution (Nano-Glo Luciferase Substrate and Nano-Glo Luciferase Assay Buffer) and detecting luminescence on a Spark multimode microplate reader (Tecan).

### *In vitro* pulldown assay

*E. coli* Lemo21(DE3) strains carrying the plasmids pSB3775, pSB4539, or pSB2835 were grown overnight and subcultured into 200 mL LB with kanamycin and chloramphenicol at 37°C until OD_600_ ≈ 0.6. Protein expression was induced with 0.5 mM IPTG, and cultures were incubated for an additional 4 h. Cells were harvested by centrifugation at 6,000 × *g* and resuspended in 5 mL lysis buffer (50 mM NaH₂PO₄, 300 mM NaCl, 10 mM imidazole, 1 mM MgCl₂, 2.5 U/mL DNase, plus cOmplete Protease Inhibitor Cocktail [Sigma 11697498001]). Lysis was performed using a One Shot cell disruptor (Constant Systems Ltd., Northants, UK), and cell debris was removed by centrifugation. The cleared lysate was incubated with 100 μL Ni-NTA resin (Qiagen 30210) for 1 h at 4°C with gentle rocking. Resin was washed four times with 5 mL ice-cold PBS + 20 mM imidazole to remove unbound proteins. Bound proteins were eluted in 200 μL PBS + 200 mM imidazole. Eluted fractions were analyzed by SDS-PAGE and Coomassie Brilliant Blue staining.

### *In vivo* photo-crosslinking

Site-specific incorporation of the photo-crosslinkable amino acid *p*-benzoyl-L-phenylalanine (*p*Bpa; Bachem) was achieved by replacing the chosen codon with a TAG stop codon in the chromosome. This chromosomal strategy avoids artifacts associated with multicopy plasmid expression. The resulting *S*. Typhimurium strains harboring TAG mutations were co-transformed with plasmids pSupBpa and pSB3292, enabling TAG suppression by *p*Bpa incorporation and boosting SPI-1 expression, respectively. Overnight cultures of these co-transformants were diluted 1:20 into LB + 0.3 M NaCl containing 50 μg/mL ampicillin, 10 μg/mL chloramphenicol, 1 mM *p*Bpa, and 0.075% arabinose, then grown at 37°C under low-aeration conditions for 6 h. Cells (1 mL) were transferred to 35-mm tissue-culture dishes (Falcon 353001) and exposed to UV light (λ = 365  nm) from a handheld lamp for 45 min. Control samples were kept in the dark. After crosslinking, cells were pelleted, resuspended in 100 μL SDS-PAGE loading buffer, and 20 μL was run on SDS-PAGE for immunoblot analysis.

### Immunodetection

Protein samples were separated by SDS-PAGE and transferred to nitrocellulose membranes, which were blocked in TBS plus nonfat dry milk. Primary antibodies used were anti-FLAG (Sigma), anti-M45, and anti-HA (BioLegend). Secondary antibodies conjugated to DyLight 800 or DyLight 680 were used to visualize bands on a Li-Cor Odyssey infrared imaging system.

### AlphaFold modeling

An AlphaFold2 (38, 39) structural model of SpaO^L^ in complex with a SpaO^short^ dimer was generated using ColabFold (39). The model with the lowest predicted alignment error (PAE) was visualized and analyzed in ChimeraX (49). Inter-residue distances were examined using the SELECT command in ChimeraX.

## Data Availability

All data are available in the main text and supplemental material.

## SUPPLEMENTAL MATERIAL

Supplemental Material mbio.00155-26-s0001.pdf Supplemental figures and tables.

ASM does not own the copyrights to Supplemental Material that may be linked to, or accessed through, an article. The authors have granted ASM a non-exclusive, world-wide license to publish the Supplemental Material files. Please contact the corresponding author directly for reuse.
