# Supplementary material for: Interplay between SpaO variants shapes the architecture of the Salmonella type III secretion sorting platform
Source: mBio. 2026 Feb 27;17(4):e00155-26. doi: 10.1128/mbio.00155-26 (PMC13059759; doi:10.1128/mbio.00155-26)
Supplement: Supplemental Material — Supplemental figures and tables. [file mbio.00155-26-s0001.pdf]

Supplemental Material

Interplay between SpaO variants shapes the architecture of the *Salmonella* type III secretion sorting platform

José Eduardo Soto[#], Tingting Wang, Jorge E. Galán*, and Maria Lara-Tejero*

Department of Microbial Pathogenesis, Yale University School of Medicine, New Haven CT06536

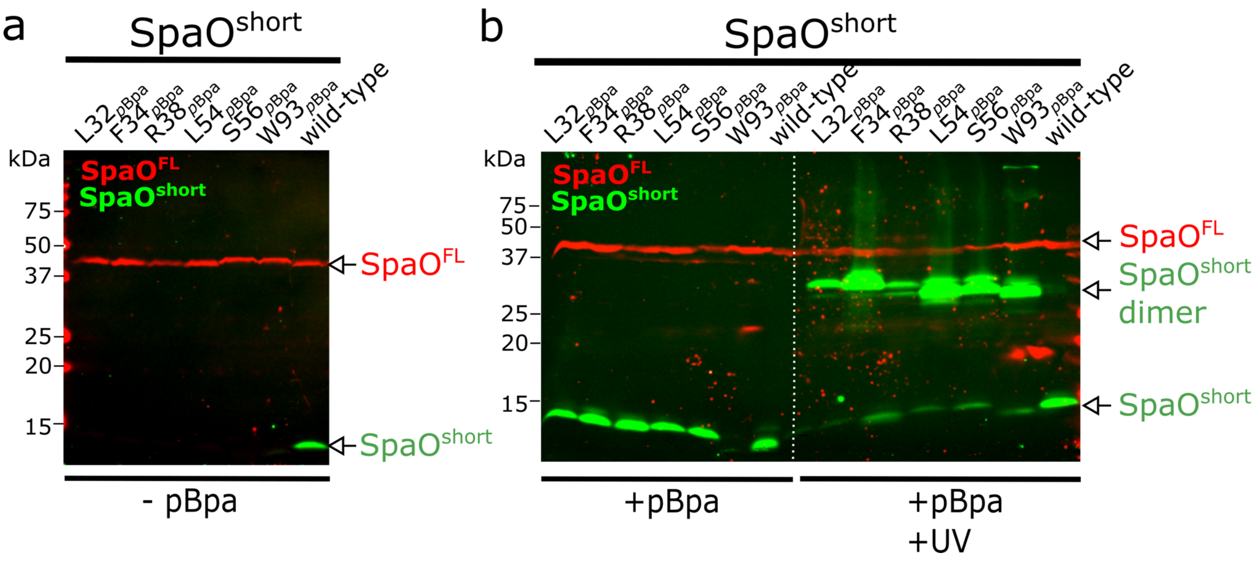

Fig S1. Controls for SpaO photo-crosslinking assays. (a and b) Immunodetection of ^{3FLAG}SpaO^{L} (red) and M45-SpaO^{short} (green) from *S*. Typhimurium uncoupled strains carrying either wild-type SpaO^{short} or the indicated SpaO^{short} *p*Bpa variants expressed from their chromosomal loci. Strains were grown in the absence (a) or presence (b) of the unnatural amino acid *p*Bpa. No *p*Bpa-containing SpaO^{short} variants were detected in the absence of *p*Bpa (a). Panel (b) shows the same western-blot as Fig. 2d displayed with increased signal intensity to highlight the lack of SpaO^{L}-SpaO^{short} crosslinked species.

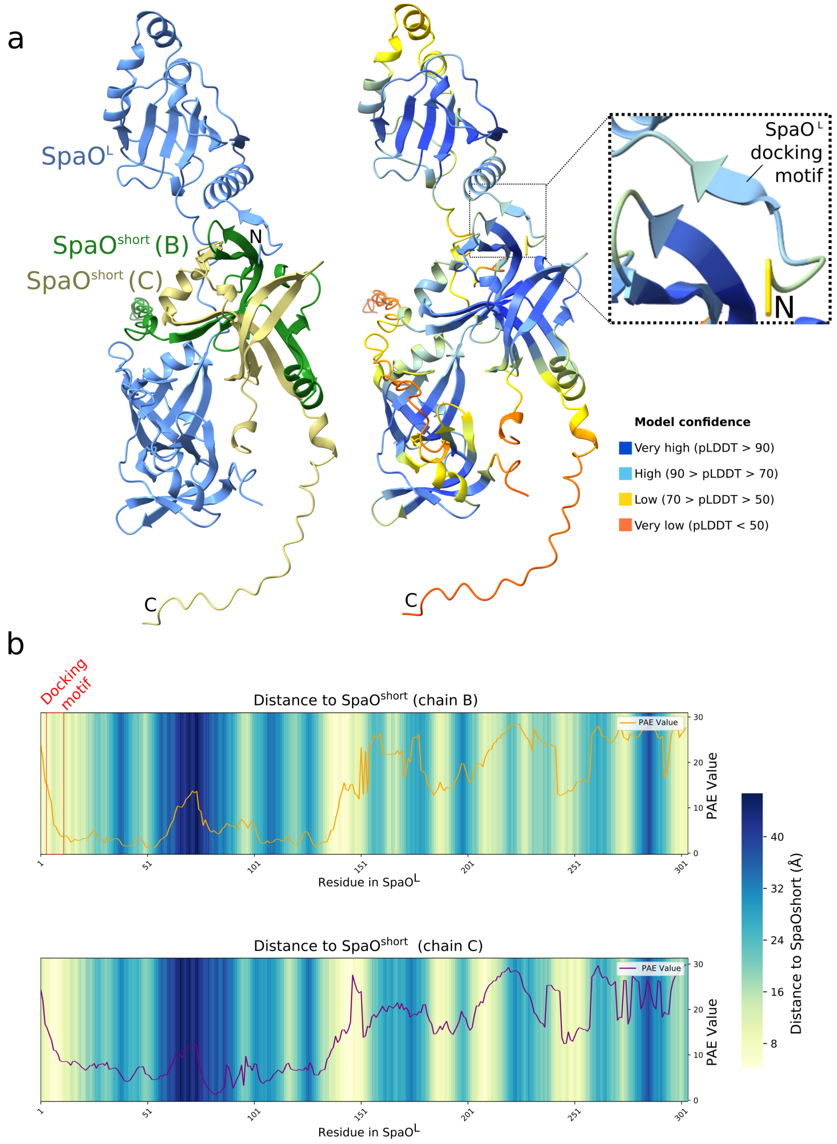

Fig S2. AlphaFold metrics for the SpaO^{L}-2SpaO^{short} complex. (a) Left, AlphaFold predicted structure of the SpaO^{L} (blue)-SpaO^{short} dimer (green and khaki) complex. Right, the AF per residue confidence score (pLDDT) was mapped onto the structure. The N- and C-terminal ends of SpaO^{L} are indicated. Inset shows zoom-in on the interface between SpaO^{L} docking motif and SpaO^{short}. (b) Heatmaps showing the distance in Å between each residue of SpaO^{L} and its nearest residue of SpaO^{short}, either in protomer B (upper panel) or C (lower panel). Predicted Aligned Error (PAE) values for the corresponding residue pairs are overlaid. Lower PAE values indicate high confidence relative positioning. Highlighted in red is the identified docking motif in the N-terminal extreme of SpaO^{L}.

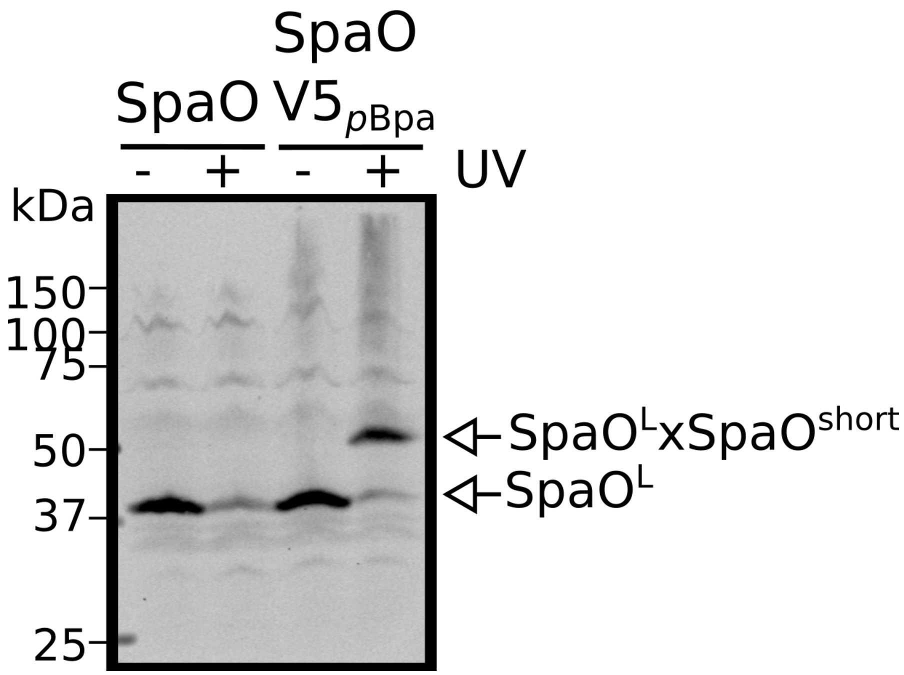

Figure S3. Capture of the SpaO^{L}-SpaO^{short} crosslinked complex within the native *Salmonella* locus. Whole cell lysates of *S*. Typhimurium chromosomally expressing either the N-terminally 2HA-tagged wild-type SpaO protein or an isogenic strain expressing the SpaOV5*p*Bpa allele were analyzed by western-blot. Samples were exposed to UV light or left untreated and probed with monoclonal antibodies against the HA epitope. The detection of the SpaO^{L} -SpaO^{short} crosslinked complex is indicated. A UV-dependent ~50-kDa SpaO-containing adduct is observed in the *p*Bpa-substituted strain, recapitulating the cross-linking pattern detected in the uncoupled, recoded strain using anti-FLAG antibodies (Fig. 3b).

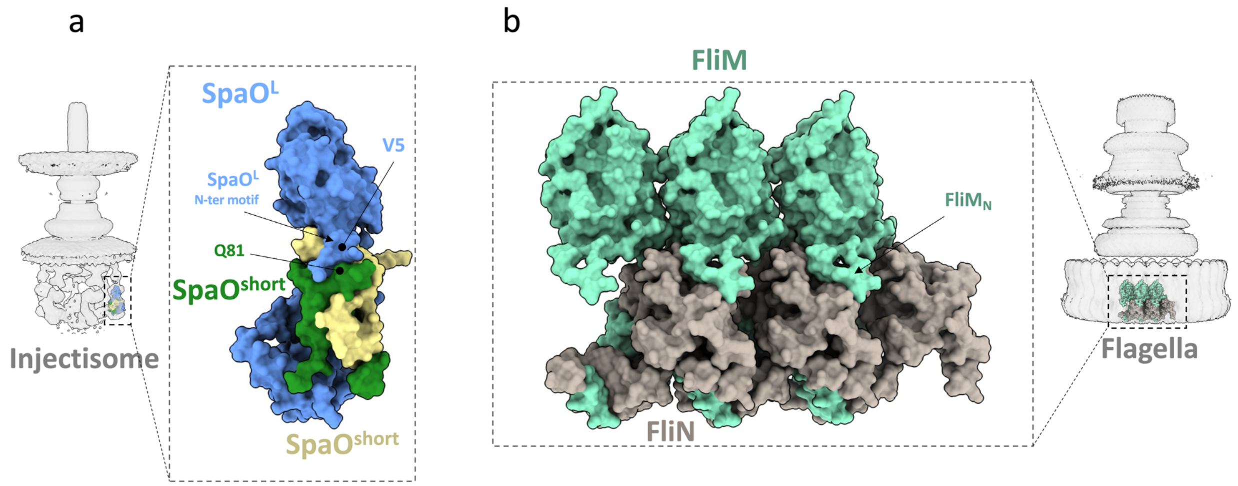

Figure S4. Structural comparison between injectisome and flagellar C-ring assembly units. (a) Model of the proposed building block of the *Salmonella* sorting platform, consisting of one SpaO^{L} molecule (blue) bound to a SpaO^{short} dimer (green and khaki). The SpaO^{L} N-terminal docking motif identified in this study is indicated, along with SpaO^{L}-V5 and SpaO^{short}-Q81 residues, which yielded the strongest photo-crosslinking signal *in vivo*. The approximate location of this SpaO-based module within the *in situ Salmonella* injectisome map (EMDB-8544) is shown for reference. (b) Solved structure of the flagellar C-ring subassembly unit (PDB: 8XP0), composed of three FliM (teal)-FliN (gray) heterotetramers 3x(1 FliM:3 FliN). The N-terminal domain of FliM (FliM$_{N}$, residues 34-45) that was reported to bind the central groove of a FliN dimer [41], is indicated. The fit of the FliM-FliN complex into the flagellar C-ring density map (EMD-37679) is shown for comparis

Table S1. Comparative nomenclature of homologous T3SS proteins among pathogenic bacteria relevant to this study, along with their corresponding flagellar homologs. SpaO family members are highlighted in yellow.

Function	Unified nomenclature	Salmonella spp. SPI-1	Shigella flexneri	Salmonella spp. SPI-2	EPEC	Yersinia spp.	Xanthomonas spp.	Flagella
**Secretin**	SctC	InvG	MxiD	SsaC	EscC	YscC	HrcC	-
**Major IM ring**	SctD	PrgH	MxiG	SsaD	EscD	YscD	HrcD	FliF#
**Minor IM ring**	SctJ	PrgK	MxiJ	SsaJ	EscJ	YscJ	HrcJ	FliF
**Export apparatus**	SctR	SpaP	Spa24	SsaR	EscR	YscR	HrcR	FliP
**Export apparatus**	SctS	SpaQ	Spa9	SsaS	EscS	YscS	HrcS	FliQ
**Export apparatus**	SctT	SpaR	Spa29	SsaT	EscT	YscT	HrcT	FliR
**Export apparatus**	SctU	SpaS	Spa40	SsaU	EscU	YscU	HrcU	FlhB
**Export apparatus**	SctV	InvA	MxiA	SsaV	EscV	YscV	HrcV	FlhA
**Symmetry adapter**	SctK	OrgA	MxiK	STM1410	EscK	YscK	HrpB4	FliG
**SP pod major component**	**$SctQ^{L}$**	**$SpaO^{L}$**	**$Spa33^{L}$**	**$SsaQ^{L}$**	**EscQ**	**$YscQ^{L}$**	**$HrcQ^{L}$**	**FliM**
**SP pod stabilizer**	**$SctQ^{short}$**	**$SpaO^{short}$**	**$Spa33^{short}$**	**$SsaQ^{short}$**	*	**$YscQ^{short}$**	**$HrcQ^{short}$**	**FliN**
**SP spokes**	SctL	OrgB	MxiN	SsaK	EscL	YscL	HrcL	FliH
**ATPase**	SctN	InvC	Spa47	SsaN	EscN	YscN	HrcN	FliI
**Central stalk**	SctO	InvI	Spa13	SsaO	EscO	YscO	HrpB7	FliJ
**Inner rod**	SctI	PrgJ	MxiI	SsaI	EscI	YscI	HrpB2	FlgB/FlgC/ FlgF/FlgG
**Needle**	SctF	PrgI	MxiH	SsaG	EscF	YscF	HrpE	FliC
**Needle length regulator**	SctP	InvJ	Spa32	SsaP	EscP	YscP	HpaC	FliK
**Needle tip**	SctA	SipD	IpaD	SseB	EspA	LcrV	-	-
**Hydrophobic pore protein**	SctE	SipB	IpaB	SseC	EspD	YopB	HrpK	-
**Hydrophobic pore protein**	SctB	SipC	IpaC	SseD	EspB	YopD	XopA	-
**Gatekeeper**	SctW	InvE	MxiC	SsaL	SepL	YopN	HpaA	-

Remote homology has been proposed between FliF and SctD protein family [1]
* No internal translation product of the enteropathogenic *Escherichia coli* protein EscQ has yet been identified.

Table S2. List of strains and plasmids used in this study.

STRAIN	RELEVANT GENOTYPE	SOURCE OR REFERENCE
SB300	Wild-type *Salmonella*	[2]
SB2130	Δ*spaO*	[3]
SB2478	spaO-3xFlag Δ$spaO^{short}$	[4]
SB2759	Δ*spaOshort*	[4]
SB2808	14xHis- 3xFlag- SpaO	[4]
SB4361	14xHis- 3xFlag- SpaOV203A M45- SpaOS	This work
SB4351	14xHis- 3xFlag- SpaOV203A, M45- SpaOshort L32 to TAG	This work
SB4169	14xHis- 3xFlag- SpaOV203A, M45- SpaOshort F34 to TAG	This work
SB4352	14xHis- 3xFlag- SpaOV203A, M45- SpaOshort R38 to TAG	This work
SB4168	14xHis- 3xFlag- SpaOV203A, M45- SpaOshort L54 to TAG	This work
SB4353	14xHis- 3xFlag- SpaOV203A, M45- SpaOshort S56 to TAG	This work
SB4170	14xHis- 3xFlag- SpaOV203A, M45- SpaOshort W93 to TAG	This work
SB4362	14xHis- 3xFlag- SpaOV203A, V5 to TAG and M45- SpaOshort	This work
SB4363	14xHis- 3xFlag- SpaOV203A, R6 to TAG and M45- SpaOshort	This work
SB4364	14xHis- 3xF- SpaOV203A, I8 to TAG and M45- SpaOshort	This work
SB4354	14xHis- 3xFlag- SpaOV203A, R10 to TAG, M45- SpaOshort	This work
SB4410	14xHis- 3xFlag- SpaOV203A, and M45-SpaOshort N70 to TAG	This work
SB4411	14xHis- 3xFlag- SpaOV203A, and M45-SpaOshort L79 to TAG	This work
SB4365	14xHis- 3xFlag- SpaOV203A, and M45- SpaOshort Q81 to TAG	This work
SB4412	14xHis- 3xFlag- SpaOV203A, and M45-SpaOshort E89 to TAG	This work
SB4292	14xHis- 3xFlag- SpaO Δ3-10	This work
SB4541	14xHis- 3xFlag- SpaO R6E	This work
SB4295	14xHis- 3xFlag- SpaOV203A Δ3-10, and M45- SpaOshort Q81 to TAG	This work
SB4296	14xHis- 3xF- SpaOV203A R6E, and M45- SpaOshort Q81 to TAG	This work
SB4367	2HA-SpaOV203A, SpaOshort-M45, OrgA-3FLAG	This work
SB4368	2HA-SpaOV203A and V5 to TAG, SpaOshort-M45, OrgA-3FLAG	This work

SB4369	2HA-SpaOV203A and T87 to TAG, SpaOshort-M45, OrgA-3FLAG	This work
SB4370	2HA-SpaOV203A, V5 and T87 to TAG, SpaOshort-M45, OrgA-3FLAG	This work
SB4401	2HA-SpaOV203A and V5 to TAG, SpaOshort-M45, OrgB-3FLAG	This work
SB4402	2HA-SpaOV203A and N285 to TAG, SpaOshort-M45, OrgB-3FLAG	This work
SB4403	2HA-SpaOV203A, V5 to TAG, and N285 to TAG, SpaOshort-M45, OrgB-3FLAG	This work
SB4433	14xHis- 3xFlag- SpaOV203A, V5 to TAG and M45- SpaOshort, F34 to TAG	This work
SB3888	orgA3XFlag, 2HA-SpaO	[5]
SB4404	orgA3XFlag, 2HA-SpaO V5 to TAG	This work
SB4405	prgHΔ100, 14xHis- 3xF- SpaOV203A, V5 to TAG and M45-SpaOshort	This work
SB4406	Δ*orgA*, 14xHis- 3xFlag- SpaOV203A, V5 to TAG and M45-SpaOshort	This work
SB4407	Δ*orgB*, 14xHis- 3xFlag- SpaOV203A, V5 to TAG and M45-SpaOshort	This work
SB4408	Δ*invC*, 14xHis- 3xFlag- SpaOV203A, V5 to TAG and M45-SpaOshort	This work
SB4409	Δ*invI*, 14xHis- 3xFlag- SpaOV203A, V5 to TAG and M45-SpaOshort	This work
E. coli β-2163 Δnic35	Strain for suicide vector delivery. Requires diaminopimelic acid	[6]
E. coli CC118 pir	Strain that expresses the π protein for replication of plasmids with the R6K origin.	[7]

PLASMID	RELEVANT GENOTYPE	SOURCE OR REFERENCE
pBAD24A	Arabinose-inducible bacterial expression vector	[8]
pSB6655	pBAD24A+sicP+sptP-NP-N7	This work
pSB3292	pBAD24A+hilA	[3]
pSup-Bpa	Suppressor plasmid to incorporate the unnatural aa pBpa	[9]

pSB3775	pET28a+14xHis-SpaO	[4]
pSB4539	pET28a+14xHis-SpaOV203A	Lara-Tejero Unpublished
pSB2835	pET28a+14xHis-SpaOΔ3-10	This work
pSB890	R6K origin, suicide plasmid counter selectable with sucrose	[10]
pSB4540	pSB890-based construct to introduce Δ*spaOshort*	[4]
pSB6786	pSB890-based construct to introduce 14xHis- 3xFlag-SpaOV203A M45- SpaOS	This work
pSB6581	pSB890-based construct to introduce 14xHis- 3xFlag-SpaOV203A, M45- SpaOshort L32 to TAG	This work
pSB6582	pSB890-based construct to introduce 14xHis- 3xFlag-SpaOV203A, M45- SpaOshort F34 to TAG	This work
pSB6583	pSB890-based construct to introduce 14xHis- 3xFlag-SpaOV203A, M45- SpaOshort R38 to TAG	This work
pSB6584	pSB890-based construct to introduce 14xHis- 3xFlag-SpaOV203A, M45- SpaOshort L54 to TAG	This work
pSB6585	14xHis- 3xFlag- SpaOV203A, M45- SpaOshort S56 to TAG	This work
pSB6586	pSB890-based construct to introduce 14xHis- 3xFlag-SpaOV203A, M45- SpaOshort W93 to TAG	This work
pSB6787	pSB890-based construct to introduce 14xHis- 3xFlag-SpaOV203A, V5 to TAG and M45- SpaOshort	This work
pSB6788	pSB890-based construct to introduce 14xHis- 3xFlag-SpaOV203A, R6 to TAG and M45- SpaOshort	This work
pSB6789	pSB890-based construct to introduce 14xHis- 3xFlag-SpaOV203A, I8 to TAG and M45- SpaOshort	This work
pSB6590	pSB890-based construct to introduce 14xHis- 3xFlag-SpaOV203A, R10 to TAG, M45- SpaOshort	This work
pSB6800	pSB890-based construct to introduce 14xHis- 3xFlag-SpaOV203A, and M45-SpaOshort N70 to TAG	This work
pSB6801	pSB890-based construct to introduce 14xHis- 3xFlag-SpaOV203A, and M45-SpaOshort L79 to TAG	This work
pSB6790	pSB890-based construct to introduce 14xHis- 3xFlag-SpaOV203A, and M45- SpaOshort Q81 to TAG	This work
pSB2824	pSB890-based construct to introduce 14xHis- 3xFlag-SpaOV203A, and M45-SpaOshort E89 to TAG	This work
pSB6264	pSB890-based construct to introduce 14xHis- 3xFlag-SpaO Δ3-10	This work
pSB6992	pSB890-based construct to introduce 14xHis- 3xFlag-	This work

	SpaO R6E	
pSB6267	pSB890-based construct to introduce 14xHis- 3xFlag-SpaOV203A Δ3-10, and M45- SpaOshort Q81 to TAG	This work
pSB6268	pSB890-based construct to introduce 14xHis- 3xFlag-SpaOV203A R6E, and M45- SpaOshort Q81 to TAG	This work
pSB6792	pSB890-based construct to introduce 2HA-SpaOV203A M45- SpaOS	This work
pSB6794	pSB890-based construct to introduce 2HA-SpaOV203A, V5 to TAG and M45- SpaOshort	This work
pSB6795	pSB890-based construct to introduce 2HA-SpaOV203A, T87 to TAG and M45- SpaOshort	This work
pSB6796	pSB890-based construct to introduce 2HA-SpaOV203A, N285 to TAG and M45- SpaOshort	This work
pSB6798	pSB890-based construct to introduce 2HA-SpaOV203A, V5 to TAG, T87 to TAG and M45-SpaOshort	This work

References

1. Bergeron, J.R., *Structural modeling of the flagellum MS ring protein FliF reveals similarities to the type III secretion system and sporulation complex.* PeerJ, 2016. **4**: p. e1718.
2. Hoiseth, S.K. and B.A. Stocker, *Aromatic-dependent Salmonella typhimurium are non-virulent and effective as live vaccines.* Nature, 1981. **291**(5812): p. 238-9.
3. Lara-Tejero, M., et al., *A sorting platform determines the order of protein secretion in bacterial type III systems.* Science, 2011. **331**(6021): p. 1188-91.
4. Lara-Tejero, M., et al., *Role of SpaO in the assembly of the sorting platform of a Salmonella type III secretion system.* PLoS Pathog, 2019. **15**(1): p. e1007565.
5. Soto, J.E., J.E. Galan, and M. Lara-Tejero, *Assembly and architecture of the type III secretion sorting platform.* Proc Natl Acad Sci U S A, 2022. **119**(51): p. e2218010119.
6. Demarre, G., et al., *A new family of mobilizable suicide plasmids based on broad host range R388 plasmid (IncW) and RP4 plasmid (IncPalpha) conjugative machineries and their cognate Escherichia coli host strains.* Res Microbiol, 2005. **156**(2): p. 245-55.
7. Herrero, M., V. de Lorenzo, and K.N. Timmis, *Transposon vectors containing non-antibiotic resistance selection markers for cloning and stable chromosomal insertion of foreign genes in gram-negative bacteria.* J Bacteriol, 1990. **172**(11): p. 6557-67.
8. Guzman, L.M., et al., *Tight regulation, modulation, and high-level expression by vectors containing the arabinose PBAD promoter.* J Bacteriol, 1995. **177**(14): p. 4121-30.
9. Ryu, Y. and P.G. Schultz, *Efficient incorporation of unnatural amino acids into proteins in Escherichia coli.* Nat Methods, 2006. **3**(4): p. 263-5.
10. Kaniga, K., J.C. Bossio, and J.E. Galan, *The Salmonella typhimurium invasion genes invF and invG encode homologues of the AraC and PulD family of proteins.* Mol Microbiol, 1994. **13**(4): p. 555-68.
